# High Interleukin 21 Levels in Patients with Systemic Lupus Erythematosus: Association with Clinical Variables and rs2221903 Polymorphism

**DOI:** 10.3390/jcm13154512

**Published:** 2024-08-02

**Authors:** Noemí Espinoza-García, Diana Celeste Salazar-Camarena, Miguel Marín-Rosales, María Paulina Reyes-Mata, María Guadalupe Ramírez-Dueñas, José Francisco Muñoz-Valle, Itzel María Borunda-Calderón, Aarón González-Palacios, Claudia Azucena Palafox-Sánchez

**Affiliations:** 1 Doctorado en Ciencias en Biología Molecular en Medicina (DCBMM), Centro Universitario de Ciencias de la Salud, Universidad de Guadalajara, Guadalajara 44340, Jalisco, Mexico; noemi.espinoza@academicos.udg.mx; 2 Grupo de Inmunología Molecular, Centro Universitario de Ciencias de la Salud, Universidad de Guadalajara, Guadalajara 44340, Jalisco, Mexico; celeste.salazar@academicos.udg.mx (D.C.S.-C.); miguel.marin@academicos.udg.mx (M.M.-R.); paulina.reyes@academicos.udg.mx (M.P.R.-M.); aaron.gonzalez@academicos.udg.mx (A.G.-P.); 3 Hospital General de Occidente, Secretaría de Salud Jalisco, Guadalajara 45170, Jalisco, Mexico; 4 Instituto de Investigación en Ciencias Biomédicas (IICB), Centro Universitario de Ciencias de la Salud, Universidad de Guadalajara, Guadalajara 44340, Jalisco, Mexico; maria.rduenas@academicos.udg.mx (M.G.R.-D.); jose.mvalle@academicos.udg.mx (J.F.M.-V.); 5 Doctorado en Ciencias Biomédicas (DCB), Centro Universitario de Ciencias de la Salud, Universidad de Guadalajara, Guadalajara 44340, Jalisco, Mexico; itzel.borunda2324@alumnos.udg.mx

**Keywords:** Interleukin 21, systemic lupus erythematosus, *IL21* polymorphism

## Abstract

**Background:** Systemic lupus erythematosus (SLE) is an autoimmune disease characterized by autoantibody production and diverse tissue and organ inflammatory affections. Interleukin 21 (IL-21) is implicated in B cell survival, proliferation, differentiation, class switching, and immunoglobulin production; therefore, it is considered a key cytokine in the pathogenesis of SLE. However, its association with disease activity and clinical phenotypes remains unclear. We aimed to evaluate the association of IL-21 levels with the disease activity and clinical phenotypes in patients with SLE. Also, we analyzed the *IL21* polymorphisms associated with increased IL-21 levels. **Methods**: The IL-21 serum levels were determined using the enzyme-linked immunosorbent assay (ELISA) method. The rs2221903 and rs2055979 polymorphisms were assessed in 300 healthy controls (HCs) and 300 patients with SLE by the polymerase chain reaction–restriction fragment length polymorphism (PCR-RFLP) technique. The levels of IL-21 were monitored during follow-up visits in 59 patients with SLE. **Results**: The patients with SLE showed higher IL-21 levels compared to the HCs. The IL-21 levels did not correlate with Mex-SLEDAI and were not different in patients with inactive, mild–moderate, and severe disease. The IL-21 levels were increased in patients with hematological affection. The ROC curve analysis revealed that the IL-21 levels had good predictive power in discriminating among patients with SLE and HCs. In a follow-up analysis, the levels of IL-21 remained higher in the patients with SLE even when the patients were in remission. Also, the rs2221903 polymorphism was associated with increased IL-21 levels. **Conclusions**: This study highlights the importance of IL-21 as a key cytokine in SLE. IL-21 levels are higher in patients with SLE and remain increased regardless of disease activity. According to the ROC analysis, IL-21 is a potential biomarker of SLE. Further longitudinal studies are needed to explore the relationship between IL-21 and the clinical phenotypes of SLE.

## 1. Introduction

Systemic lupus erythematosus (SLE) is a chronic autoimmune disease characterized by autoantibody production, immune complex formation, and inflammatory tissue damage [1]. In the SLE pathogenesis, diverse cytokines are involved in the onset, progression, and exacerbation of the disease, including interferon-alpha (IFNα), tumor necrosis factor-alpha (TNFα), interleukin (IL) 6, IL-10, IL-12, IL-17, IL-21, IL-27, and B cell-activating factor (BAFF), among others [2,3,4,5,6], highlighting SLE as a complex multi-cytokine disease.

IL-21 is a pleiotropic cytokine produced by T follicular helper (Tfh) cells, circulating Tfh (cTfh), T peripheral helper (Tph) cells, T helper 17 (Th17), T helper 9 (Th9) cells, and Natural Killer T cells [7,8,9,10]. These cells can help B cells through IL-21 [9], promoting B cell activation and differentiation, affinity maturation, class switching, and antibody production [11,12,13]. It has been reported that IL-21 levels are increased in patients with SLE compared to controls [4,14,15]. To our knowledge, lupus nephritis is the only clinical phenotype associated with IL-21 levels [15]. However, studies show heterogeneous results regarding the correlation between IL-21 levels and clinical variables in patients with SLE [16,17]. In a previous study, we found that patients with SLE have increased frequencies of IL-21+ cTfh and Tph cells, which are maintained independently of the disease activity [18]. The crucial role of IL-21 in SLE development is underscored by its elevated levels in affected patients compared to controls, and its persistence at high levels further highlights its significance.

Among the factors associated with the disease, genetic factors, including single-nucleotide polymorphisms (SNPs), have a special contribution. The SNPs rs2221903 (+3268 T>C) and rs2055979 (+1439 C>A), localized in the second intron of the *IL21* gene, have been associated with increased IL-21 levels [4,19]. Both polymorphisms have been associated with autoimmune diseases, such as rheumatoid arthritis [20] and multiple sclerosis [21,22]. In the context of SLE, the rs2221903 and rs2055979 SNPs have been studied by several research groups with discordant results [4,19,23].

This study aimed to evaluate the IL-21 levels in patients with SLE and compare them with healthy controls, as well as to analyze their association with disease activity, clinical phenotype, and *IL21* polymorphisms (rs2221903 and rs2055979).

## 2. Materials and Methods

### 2.1. Subjects

This study included 600 subjects: 300 HCs (273 females and 27 males) and 300 patients with SLE (283 females and 17 males). The patients with SLE were classified according to the American College of Rheumatology’s 1997 revised criteria [24] and were recruited through consecutive non-randomized selection methods in the rheumatology department of the Hospital General de Occidente, Guadalajara, Mexico. The Mexican versions of the Systemic Lupus Disease Activity Index (Mex-SLEDAI) [25] and the Systemic Lupus International Collaborating Clinics (SLICC) damage index [26] scores were applied to all patients with SLE at the moment of inclusion. The exclusion criteria were patients with overlap syndromes, pregnancy, biological therapy, and current infection. Subjects included as HCs were recruited through clinical assessments under protocols of blood donation in a blood bank, excluding subjects with chronic diseases; also, all subjects included were similar to the patients with SLE in age and gender, and they were all unrelated individuals with no first-degree family suffering from some autoimmune disease. All participants were Mexican mestizos from western Mexico [27]. The clinical activity groups for the patients with SLE were stratified according to the Mex-SLEDAI score as follows: inactive (0–1), mild–moderate (2–6), and severe (≥7) disease. Most patients were undergoing pharmacological treatment; however, none were undergoing biological therapy. In addition, a follow-up analysis was performed on 59 patients with SLE.

This study was approved by the Ethics and Research Committee from Hospital General de Occidente (no. CEI-146/21 and no. CI-146/21). Before inclusion, all participants were required to sign an informed consent form. The present study was carried out following the ethical standards and principles established in the Declaration of Helsinki and the research committees from the participant institutions [28].

### 2.2. Quantification of IL-21 Serum Levels and Anti-dsDNA Antibodies

Serum was obtained from peripheral blood samples from patients with SLE and HCs and stored at −20 °C until use. The IL-21 levels were determined in 278 patients with SLE and 170 HC using an ELISA assay (ELISA MAX™ Deluxe Set Human IL-21: cat. No 433804, BioLegend, CA, USA), performed following the manufacturer’s instructions. The ELISA kit sensitivity is 16 pg/mL, and the detection limit range is 31.3–2000 pg/mL. Samples were analyzed undiluted in duplicate and read at 450 and 570 nm using the Multiskan™ Go Microplate Spectrophotometer (Thermo Fisher Scientific, Waltham, WA, USA). The results of anti-double-stranded DNA (anti-dsDNA) antibody test were taken from medical records. The anti-dsDNA antibody test was performed by Crithidia luciliae indirect immunofluorescence method.

### 2.3. Genotyping of IL21 rs2221903 and rs2055979 Polymorphisms

Genomic DNA (gDNA) was purified from peripheral blood samples of patients with SLE and HCs using the modified Miller’s technique [29]. The rs2221903 and rs2055979 polymorphisms were genotyped using the polymerase chain reaction–restriction fragment length polymorphism (PCR-RFLP) technique. The amplification of the DNA fragment containing the rs2221903 (+3268 T>C) polymorphism was performed using the following primers: forward: 5′-TGGACACTGACGCCCATATTGA-3′ and reverse: 5′-AAG GCAGTTTAGTGGCGACAGC-3′. For the rs2055979 (+1439 C>A) SNP, the following primers were used: forward: 5′-CAG CCA GGA AAC TCT GGA AAG AA-3′ and reverse: 5′-GCTCTGAACCCAAACACTCTCATTT-3′ [4]. Both PCRs were carried out in a total volume of 25 μL containing the following: 1X PCR buffer, 4 mM MgCl_2_, 2.5 mM dNTPs, 2 μM of each primer, 0.5 units of Taq DNA polymerase (Invitrogen Life Technologies, Carlsbad, CA, USA), and 100 ng of gDNA. The PCR cycling conditions used were as follows: initial denaturation cycle at 95 °C for 5 min, followed by 32 cycles of denaturation for 50 s at 95 °C, annealing for 30 s at 65 °C, extension for 30 s at 72 °C, and final extension for 5 min at 72 °C.

The amplified 230 bp PCR product of the rs2221903 (+3268 T>C) polymorphism was subjected to digestion with 3 IU of the *MboII* restriction enzyme (New England BioLabs^®^, Ipswich, MA, USA) for 1 h at 37 °C. The resulting restriction fragments based on the genotype were TT: 230 bp; TC: 230, 149, and 81 bp; and CC: 149 and 81 bp. As for the rs2055979 (+1439 C>A) polymorphism, the 212 bp PCR product was digested using 5 IU of *NIaIII* restriction enzyme (New England BioLabs^®^, Ipswich, MA, USA) for 1 h at 37 °C. In this case, the restriction fragments and genotypes were CC: 158 and 54 bp; CA: 212, 158, and 54 bp; and AA: 212 bp. The digested PCR products were resolved in 6% polyacrylamide gels and stained with AgNO_3_.

### 2.4. Statistical Analysis

The data were analyzed using software packages from IBM SPSS statistics v25 (IBM Corporation; Armonk, NY, USA) and GraphPad Prism v10.2.3 (GraphPad Software Incorporation; La Jolla, CA, USA). The Kolmogorov–Smirnov test was used to assess variable distribution. Categorical variables are presented as absolute values and percentages, whereas continuous variables are presented as medians and 25th–75th percentiles. According to the case, the Kruskal–Wallis test, post hoc Dunn’s test, Mann–Whitney U test, and Wilcoxon test were used to compare groups. The Receiver Operator Characteristic (ROC) curve analysis was used to evaluate the IL-21 sensitivity and specificity in discriminating between patients with SLE and controls. The Chi-square test was used to calculate the Hardy–Weinberg equilibrium. Genotypic and allelic frequencies of *IL21* polymorphisms were determined by direct counting, and comparison was performed using the Chi-square test or Fisher’s exact test. The Odds Ratios (ORs) and 95% confidence intervals (95% CI) were calculated to determine the risk of SLE associated with the *IL21* SNPs. The haplotype inference was calculated by the EM algorithm and the SHEsis software platform [30,31]. The *p*-value was adjusted using Bonferroni correction when appropriate, and a *p*-value < 0.05 was considered with statistical significance.

## 3. Results

### 3.1. Subjects’ Demographic and Clinical Characteristics

The demographic and clinical characteristics of patients with SLE are summarized in Table 1. The median ages were 35 [interquartile range (IQR) 25–48] years old for patients with SLE and 29 (IQR 25–38) years old for HCs. The median of disease evolution was 4 (IQR 1.4–11) years for patients with SLE. Eighty-one percent of patients with SLE had inactive or mild–moderate disease activity according to Mex-SLEDAI, with a median score value of 2 (IQR 1–6); additionally, most patients had no damage with a median score of 0 (IQR 0–1) according to the SLICC damage index. Concerning treatment, prednisone was prescribed in 68% with a median dosage of 10 (IQR 5–20) mg/day, followed by antimalarial drugs (58.3%) and azathioprine (48%).

### 3.2. Association of IL-21 Levels with Clinical Phenotype of Patients with SLE

**Figure 1:**
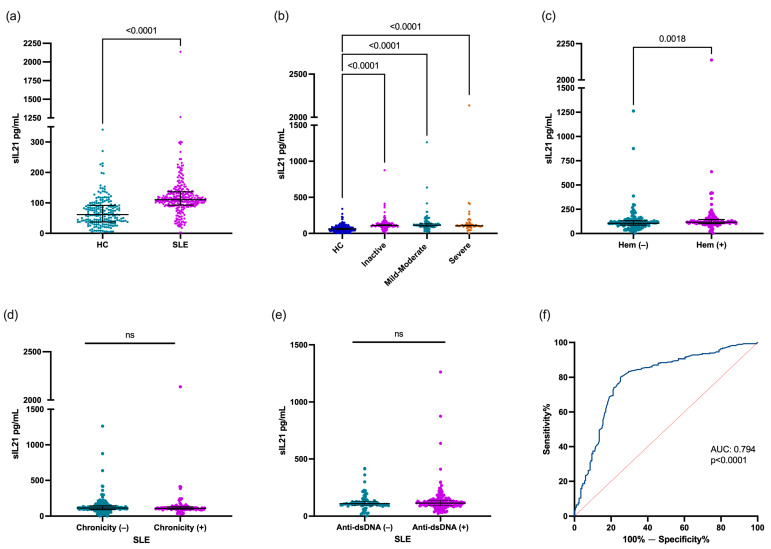
Evaluation of IL-21 levels according to clinical characteristics in patients with SLE. Comparison of IL-21 levels between HCs and patients with SLE (**a**), IL-21 levels according to disease activity (**b**), hematological domain (**c**) and chronicity (**d**), IL-21 levels according to anti-dsDNA status (**e**), and IL-21 performance as biomarker diagnosis in SLE (**f**). Patients with SLE were stratified according to Mex-SLEDAI score as follows: inactive (0–1), mild–moderate (2–6), and severe (≥7) disease. Hematological domain (Hem) included lymphopenia (<1.2 × 10^3^/µL), leukopenia (<4.0 × 10^3^/µL), thrombocytopenia (<100 × 10^3^/µL), and hemolytic anemia. Data are shown as median and IQR. *p*-value was obtained through Mann–Whitney U test, Kruskal–Wallis test with Dunn’s post hoc test, and Spearman’s correlation test, according to case. Area Under the Curve was calculated through ROC curves. ns, no significative.

The IL-21 levels were higher in patients with SLE [110.5 (IQR 92.7–136.5) pg/mL] compared with the HCs [61.7 (IQR 37.4–91.6) pg/mL; *p* < 0.0001. Figure 1a)]. The IL-21 levels were compared according to the clinical phenotype of patients with SLE. All patients with SLE with inactive disease, mild–moderate disease activity, and severe disease activity had higher IL-21 levels in comparison with the HCs [109.4 (IQR 92.7–91.6) pg/mL, 113.1 (IQR 91.6–137.0) pg/mL, and 105.7 (IQR 94.9–123.3) pg/mL vs. 61.7 (IQR 37.4–91.6) pg/mL, respectively; *p* < 0.0001; Figure 1b]. However, according to the disease activity, no significant differences were observed in the IL-21 levels between patients with SLE. When the patients with SLE were stratified according to clinical domains, the patients with SLE with hematological affection showed higher levels of IL-21 vs. no hematological affection [116.7 (101.5–143.8) pg/mL vs. 105.7 (87.4–131.0) pg/mL; *p* = 0.0018; Figure 1c], and other clinical domains did not show statistical difference (*p* > 0.05). Also, when the patients with SLE were classified according to chronicity [chronicity (−) 113.1 (IQR 90.5–143.0) pg/mL vs. chronicity (+) 108.9 (IQR 98.5–129.3) pg/mL; *p* = 0.8213; Figure 1d)] and anti-dsDNA status [anti-dsDNA (−) 108.9 (IQR 95.5–130.3) pg/mL vs. anti-dsDNA (+) 114–1 (IQR 92.7–139.2) pg/mL; *p* = 0.5209; Figure 1e], no statistical difference was found. On the other hand, the IL-21 levels did not correlate with the Mex-SLEDAI score or anti-dsDNA concentration (*p* > 0.05).

An ROC curve analysis was conducted to evaluate the ability of the IL-21 levels to distinguish between patients with SLE and HCs. The AUC was 0.794, meaning this cytokine showed good predictive power in discriminating between patients with SLE and controls (*p* < 0.0001, Figure 1f).

**Figure 2:**
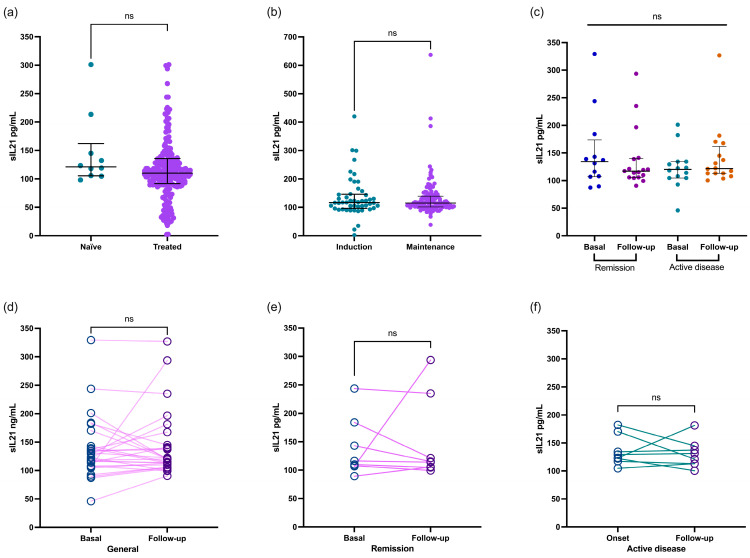
Comparison of IL-21 levels in patients with SLE based on type of treatment. Patients with SLE who were either treatment-naïve or treated showed similar concentrations of IL-21 (**a**) regardless of whether they were undergoing induction or maintenance treatment (**b**). IL-21 level comparison between patients with SLE at basal and follow-up recruitment stratified according to remission and active disease (**c**). Paired comparison of IL2-1 in patients with SLE at baseline and follow-up (**d**), as well as stratified by remission (**e**) and active (**f**) disease groups. Data are shown as median and IQR. *p*-value was obtained through Mann–Whitney U test and Wilcoxon test according to case. ns, no significative.

Regarding treatment, the IL-21 cytokine levels in the patients with SLE showed no significant difference between those receiving treatment and the untreated patients. The concentration remained consistent at 121.3 (IQR 105.5–162.1) pg/mL for the untreated patients and 115.1 (IQR 104.7–139.6) pg/mL for the treated patients (*p* = 0.4019, Figure 2a). This finding was similar even in patients undergoing the induction treatment concerning those in the maintenance phase [117.1 (IQR 96.6–145.9) vs. 115.1 (IQR 101.5–139.2) pg/mL, respectively; *p* = 0.9831; Figure 2b].

Finally, we analyzed the IL-21 levels in 59 patients with SLE during recruitment and the follow-up visits. The patients were categorized according to whether they had remission or active disease. Interestingly, the concentration of this cytokine remained similar in both groups (as shown in Figure 2c; *p* > 0.05). Even when we compared the data between the paired analyses, the IL-21 levels remained consistent regardless of whether the patients were in remission or had active disease (Figure 2d,e; *p* > 0.05).

### 3.3. Genotype and Allele Frequencies of rs2221903 and rs2055979 Polymorphisms

The genotype and allele frequency distribution of the rs2221903 and rs2055979 polymorphisms in the patients with SLE and HCs are shown in Table 2. Both polymorphisms were in Hardy–Weinberg equilibrium, with similar observed and expected frequencies in the HCs (*p* > 0.05). There were significant differences in the genotype and allele frequencies of the rs2221903 polymorphism between the patients with SLE and HCs, with a higher proportion of the C allele observed in the SLE group. According to this, the C allele as well as the TC and CC genotypes from the rs2221903 polymorphism were associated with a higher risk of SLE (OR = 1.75, 95% CI, 1.17–2.61, and *p* = 0.005; OR = 1.58, 95% CI, 1.02–2.46, and *p* = 0.039; and OR = 6.56, 95% CI, 1.07–75.60, and *p* = 0.046, respectively). In addition, the dominant model from the rs2221903 polymorphism was associated with increased SLE susceptibility (TT vs. TC+CC, OR = 1.71, 95% CI 1.11–2.62, *p* = 0.014). On the other hand, the genotype and allele frequencies of the rs2055979 polymorphism observed in the patients with SLE were not significantly different from those of the HCs.

### 3.4. Haplotype Analysis

The rs2221903 and rs2055979 polymorphisms showed a strong linkage disequilibrium (D’ = 0.88, r’ = 0.047, *p* = 0.01). As shown in Table 2, the CC haplotype was associated with increased SLE susceptibility (OR = 1.87, 95% CI, 1.21–2.85, *p* = 0.004).

### 3.5. Association of rs2221903 and rs2055979 Polymorphisms with IL-21 Levels, SLICC Damage Index, and Anti-dsDNA Antibodies

**Figure 3:**
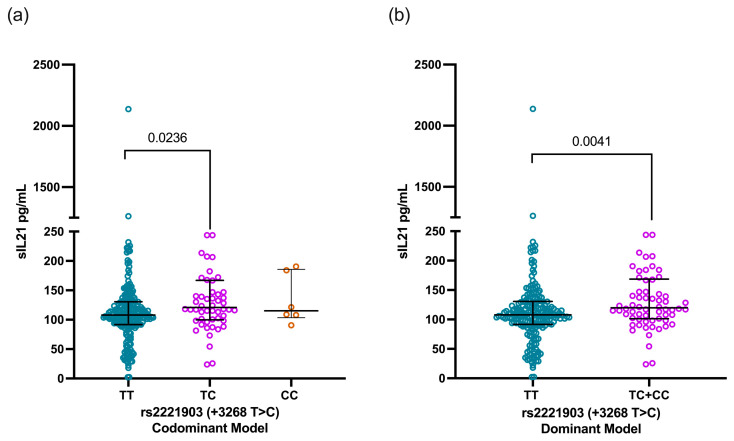
Comparison of IL-21 levels based on codominant and dominant models of rs2221903 polymorphism (+3268 T>C) in *IL21* gene. IL-21 levels according to codominant model (**a**) and dominant model (**b**) of rs2055979 polymorphism of *IL21* gene. Data are shown as median and IQR. *p*-value was obtained through Mann–Whitney U test and Kruskal–Wallis test with Dunn’s post hoc test according to case. IQR, interquartile range; HCs, healthy controls; SLE, systemic lupus erythematosus.

According to the rs2221903 polymorphism, SLE carriers of the CT genotype showed higher IL-21 levels [120.7 pg/mL (IQR 99.8–166.9)] than carriers of the TT genotype [107.8 ng/mL (91.6–130.5)] with a statistical difference (*p* = 0.0236, Figure 3a). This finding was consistent when comparing the IL-21 levels through the dominant model [TT vs. TC+CC, 107.8 pg/mL (IQR 91.6–130.5) vs. 118.2 pg/mL (IQR 101.5–167.6), *p* = 0.0041, Figure 3b)]. In contrast, the IL-21 levels were similar when analyzed according to the rs2055979 genotypes as well as for the rs2055979 and rs2221903 haplotypes (Figure S1).

## 4. Discussion

SLE is an autoimmune disorder characterized by autoantibody production and multiorgan affection [1]. The SLE pathogenesis involves different factors; nevertheless, the aberrant expression of different cytokines plays an important role in the disease’s onset, establishment, and propagation [2,3,4,5,6]. IL-21 is an important cytokine produced by different T cell subpopulations such as Tfh, cTfh, Tph, and Th17 cells. Experimentally, it has been proven that IL-21 is necessary for B cell expansion, class switching, and plasma cell development during lupus-like onset in animal models [32,33]. IL-21 in vitro stimulation, along with costimulatory signaling, increases the proportion of memory and plasma B cells [34]. Also, increased Tph cells and IL-21 have been associated with extrafollicular B cell activation and auto-antibody production [35]. In patients with SLE, Tfh cells and activated B cells were positively correlated with the IL-21 levels [16]. The blockade of IL-21 reduces dsDNA autoantibodies and total IgG as well as immunoglobulin deposits in mice [36]. IL-21 has been linked to lupus nephritis due to its role in enhancing antibody production through T cell-dependent B cell stimulation [15,37]. The above highlights the importance of IL-21 and underscores the relevance of monitoring its levels in patients with SLE. This approach provides insights into how serum cytokines can be linked to the clinical aspects of the patient.

Patients with SLE in this study showed increased IL-21 levels compared with the HCs (110.5 pg/mL vs. 61.7 pg/mL, *p* < 0.0001). There were no significant correlations between the levels of IL-21 and disease activity evaluated by the Mex-SLEDAI index, and inactive patients showed similar IL-21 levels to those with mild–moderate and severe disease. The correlation between IL-21 and disease activity is highly heterogeneous in the literature. Some studies have identified a significant association between IL-21 and high disease activity [15,16,38], whereas others found no significant relationship between IL-21 levels and disease activity [14,17,39,40]. The inconsistent results regarding the IL-21 levels and their association with disease activity may be attributed to the heterogeneity in the clinical features of patients with SLE, including the disease evolution, level of disease activity, as well as different stratifications of disease activity indices.

While the IL-21 levels are not definitively linked to disease activity, they have been found to be higher in patients with SLE. This suggests that IL-21 may play a role in the initial development and onset of SLE, impacting clinical symptoms at diagnosis and during periods of high disease activity. However, its expression appears to be unrelated to clinical manifestations in long-term cases of established SLE.

It is important to evaluate the possible biomarkers in SLE according to clinical phenotype. Therefore, we analyzed IL-21 in the group of patients according to clinical phenotypes. We found that patients with SLE with hematological involvement had higher IL-21 levels than their counterparts. The main hematological affections in our patients were cytopenias. Regarding this, it is possible that IL-2 deficiency, induced by lymphopenia, reduces the expansion and maintenance of Tregs and therefore favors greater proliferation of effector T cells and increasing IL-21 levels. IL-21, in turn, promotes the activity of effector T cells and counteracts the suppression of Tregs, creating a positive feedback loop that exacerbates immune dysregulation [41]. Previously, a single study associated IL-21 levels with lupus nephritis [15]. Our study did not find differences between IL-21 levels and other clinical domains, nor did it find differences in the damage index (chronicity). However, there were only 19 patients with lupus nephritis included in this study; therefore, our results might not be representative. The clinical phenotype is crucial for understanding biomarkers in patients with SLE. Unfortunately, our study included a heterogeneous mix of clinical phenotypes, making it difficult to compare subcategories due to the small number of patients in each group. Additionally, most reports in the literature do not specify the proportion of clinical phenotypes, further complicating comparisons.

As mentioned before, IL-21 plays a crucial role in IL-21R-expressing B cells by facilitating the activation, class switching, and differentiation of B cells for antibody production. In the context of SLE, it promotes the production of autoantibodies such as anti-dsDNA among others [32,33]. The IL-21 levels were not different among patients with positive anti-dsDNA antibodies versus those with negative anti-dsDNA antibodies. Similarly, other groups that studied patients with SLE reported a lack of association of IL-21 levels and anti-dsDNA antibodies [15,40]. In contrast, B cells from patients with SLE highly express the IL-21 receptor and respond to IL-21 in vitro to produce higher antibody levels; moreover, the IL-21 receptor correlates with anti-dsDNA antibodies [42]. Autoantibody production is a complex process that involves cytokines and costimulatory molecules through follicular and extrafollicular T cell and B cell interaction.

Another important cytokine for antibody production is BAFF, which is also correlated with IL-21 levels in patients with SLE [15]. Therefore, even when IL-21 levels are not correlated with antibody production, its function could be observed through the IL-21 receptor and other molecules involved in antibody production. It is possible that during the onset of the disease, IL-21 is positively correlated with antibody production. According to this hypothesis, in newly diagnosed Sjogren’s syndrome, the IL-21 levels correlate with the total IgG levels [43]; this association could be due to different molecular pathogenesis among different autoimmune diseases or, again, due to recently diagnosed Sjogren’s syndrome compared to patients with long-term SLE. A limitation in our study is that anti-dsDNA antibodies were qualitatively assessed; therefore, we cannot assure the lack of association between anti-dsDNA antibodies and IL-21 levels. It would be of great interest to quantitatively measure the anti-dsDNA antibodies and analyze other molecules and cytokines involved in autoantibody production, such as IL-21R and BAFF.

In 59 patients with SLE, the IL-21 levels were measured in a follow-up visit. The analysis showed that IL-21 is stable throughout time regardless of remission or active disease. In the study by Reynolds et al., in patients with long-evolution SLE disease, the IL-21 levels are stable in a follow-up quantification (around 5 months) [17]. The clinical characteristics of the patients are similar between our patients and those in their report. We previously found that IL-21-producing cTfh and Tph cells are higher in patients with SLE compared to the controls and that there were no differences according to disease activity [18]. Therefore, the expression of IL-21 seems to be constant in patients with long-term SLE disease. We further analyzed whether the IL-21 levels could be different according to the treatment of patients with SLE. The IL-21 levels did not show a statistical difference between the treated and untreated patients. In an experimental SLE treatment model, it was found that glucocorticoid reduces IL-21 expression and Tfh cells [44]. Tfh cells from patients with SLE reduce IL-21 production after glucocorticoid treatment in vitro [45]. Induction therapy in patients with untreated lupus nephritis reduced the IL-21 levels [37]. Also, in a cohort of patients with recently diagnosed myasthenia gravis, glucocorticoid treatment reduced the IL-21 levels and *IL21* mRNA in PBMC [46]. In patients with new-onset SLE, the treatment reduced Tfh cells and IL-21 levels [16]. The scenarios described above include new-onset cases of autoimmune diseases (both patients and experimental models) and in vitro stimulation. These results align with our findings and reinforce the hypothesis that there are differences in biomarkers according to disease evolution: new-onset or highly active patients versus long-term established SLE. Specifically, new-onset patients exhibited variable IL-21 levels based on treatment and disease activity, whereas patients with long-term evolution maintained higher IL-21 levels. Unfortunately, other reports of IL-21 levels do not compare treated and untreated patients, which would help support our findings. Additionally, the treatment is taken as a whole given the heterogenous prescription of patients. It would be of great interest to classify patients to compare types of treatments. Further longitudinal studies that include IL-21+ T cell populations with new patients and an extended follow-up are needed to understand IL-21’s role in the SLE pathogenesis and determine its clinical utility as a biomarker.

Finally, we evaluated the association of the *IL21* gene SNPs (rs2221903 and rs2055979) and IL-21 levels in SLE. Our results show that the C allele of the rs2221903 polymorphism is associated with SLE susceptibility in the Mexican population (OR = 1.75). Also, the rs2221903 polymorphism was associated with higher IL-21 levels. To the best of our knowledge, this is the first report that shows an association between the *IL21* gene rs2221903 polymorphism with SLE susceptibility and increased IL-21 levels in the Mexican population. The role of intronic SNPs is not well defined, but it is reported to be associated with functional consequences as they may influence mRNA translation [47]. However, other reports have failed to find an association between IL-21 levels and the rs2221903 polymorphism in patients with SLE [4,38]. On the other hand, the rs2055979 polymorphism was not associated with IL-21 levels or disease clinical variables in SLE.

Previously, the rs2221903 polymorphism has been associated with an increased risk of SLE in the Chinese, European American, African American, and Caucasian populations [19,23,48] but not in other populations such as Hispanic, Gullah, and Egyptian, as well as in another Chinese study [4,23,38]. Also, we did not find any association between the rs2055979 polymorphism and SLE, which is congruent with the findings of Sawalha et al. for European American, African American, Hispanic American, and Gullah populations [23]. However, a study in the Chinese population found an association between polymorphisms and SLE [4]. These discrepancies could be explained in part by the genetic ancestry of the studied populations. The genetic ancestry of the Mexican population from western Mexico is a mixture of European (64.6%), Native American (30.8%), and African (≈8%) ancestries [49]. Also, the Mexican mestizo population has a small proportion of Asian ancestry (1–1.4%) [49].

In addition, we found a strong linkage disequilibrium between the rs2221903 and rs2055979 polymorphisms, which was similar to that previously reported in the Chinese and Mexican populations [4,20]. The TC and TA haplotypes were the most frequent in both the HCs and patients with SLE, whereas the CC haplotype was more frequent in the SLE group. We found that the CC haplotype was associated with an increased risk for SLE (OR = 1.87, 95% CI, 1.21–2.85, *p* = 0.004), which was also similar to the finding reported by Ding et al. in a Chinese population [48]. A meta-analysis analyzed seven articles with heterogeneous results and finally pointed out that the rs2221903 CC genotype is associated with SLE risk [19]. Also, the A allele of rs2055979 was associated with SLE risk [4]; however, in another study, this polymorphism was not associated with SLE risk [23]. Therefore, more research is needed to conclude the association of these SNPs with SLE.

Our findings highlight the importance of IL-21 in the SLE pathogenesis; however, further longitudinal studies are needed to define the role of IL-21 in patients with SLE according to the remission and exacerbation of the disease activity, considering the different clinical phenotypes. Furthermore, it would be interesting to analyze the expression of intracellular IL-21 in T cell subpopulations, including IL-21 expression in the affected tissues, to elucidate IL-21’s molecular mechanisms that are directly involved in SLE. A limitation of this study is that mRNA expression was not evaluated; therefore, we do not know the relationship between gene expression and cytokine levels. The polymorphisms rs2221903 and rs2055979 exhibit heterogeneous results across various reports regarding their association with the disease, potentially due to ethnicity-specific outcomes or the presence of other polymorphisms in proximity that are yet to be discovered. According to our study, in the Mexican mestizo population, the rs2221903 polymorphism is associated with increased IL-21 levels and SLE susceptibility, while rs2055979 is not associated with these.

Further studies are still necessary to obtain a better understanding of IL-21’s role in SLE, the expression throughout the disease evolution, the association of IL-21 levels with clinical variables in SLE in longitudinal studies, and the action of its polymorphism to the risk of SLE.

## 5. Conclusions

This study highlights the importance of IL-21 as a key cytokine in SLE. The IL-21 levels are higher in patients with SLE and remain increased regardless of disease activity. The rs2221903 polymorphism of the *IL21* gene is associated with higher IL-21 levels and increased susceptibility to SLE in the Mexican population. According to the ROC analysis, IL-21 is a potential biomarker of SLE. Further longitudinal studies are needed to explore the relationship between IL-21 and the clinical phenotypes of SLE.

## Figures and Tables

**Table 1:** Demographic and clinical characteristics in patients with SLE.

Variables	SLE (*n* = 300)
Demographic features	
Age, years; median (p25–p75)	35 (25–48)
Gender (F/M)	283/17
Disease features	
Disease duration, years; median (p25–p75)	4 (1.4–11.0)
Mex-SLEDAI score; median (p25–p75)	2 (1–6)
Inactive, *n* (%)	123 (41.0)
Mild–moderate, *n* (%)	124 (41.3)
Severe, *n* (%)	53 (17.7)
SLICC score; median (p25–75)	0 (0–1)
Non-damage, *n* (%)	201 (67.0)
Damage, *n* (%)	99 (33.0)
Clinical domain	
Hematologic ^†^, *n* (%)	134 (44.7)
Mucocutaneous ^‡^, *n* (%)	102 (34.0)
Constitutional ^§^, *n* (%)	76 (25.3)
Renal ^¶^, *n* (%)	66 (22.0)
Musculoskeletal ^††^, *n* (%)	51 (17.0)
Neuropsychiatric ^‡‡^, *n* (%)	19 (6.3)
Serosal ^§§^, *n* (%)	12 (4.0)
Treatment	
Prednisone, *n* (%)	204 (68.0)
rednisone dose; median (p25–p75)	10 (5.0–20.0)
Antimalarial, *n* (%)	175 (58.3)
Azathioprine, *n* (%)	144 (48.0)
Methotrexate, *n* (%)	55 (18.3)
Mycophenolate Mofetil, *n* (%)	28 (9.3)
Cyclophosphamide, *n* (%)	27 (9.0)
Autoantibodies	
Antinuclear antibodies, *n* (%)	277/287 (96.5)
Anti-dsDNA, *n* (%)	180/268 (67.2)
Anti-RNP, *n* (%)	45/122 (36.9)
Anti-Ro, *n* (%)	40/123 (32.5)
Anti-Sm, *n* (%)	32/141 (22.7)
Anti-La, *n* (%)	17/128 (13.3)
Biochemical analysis	
Glucose (mg/dL)	91 (39.0–383.0)
Serum creatinine (mg/dL)	0.9 (0.1–10.2)
Serum urea (mg/dL)	36.5 (1.5–2.7)
Blood cell count	
Hemoglobin	12.6 (4.1–19.8)
Hematocrit	38.6 (8.3–58.1)
Leukocytes	5.7 (4.3–7.45)
Lymphocytes	1.3 (0.8–1.9)
Neutrophils	3.8 (2.6–5.2)
Platelets	243 (188–294)
ESR (mm/h)	33.0 (1.0–135.0)

The data are shown as the median and p25–p75; Mex-SLEDAI: inactive (score of 0–1), mild–moderate (score 2–6), or severe (≥7); SLICC: non-damage (SLICC score of 0) or damage (SLICC score > 1); ^†^ hematologic: leukopenia, lymphopenia, and thrombocytopenia; ^‡^ mucocutaneous: malar rash, alopecia, oral ulcers, and photosensitivity; ^§^ constitutional: fatigue; ^¶^ renal: persistent proteinuria (>0.5 g/day) and cellular casts; ^††^ musculoskeletal: articular involvement; ^‡‡^ neuropsychiatric: neurologic damage, psychosis, and convulsions; ^§§^ serosal: Raynaud’s phenomenon and serositis. SLE, Systemic Lupus Erythematosus; Mex-SLEDAI, Mexican version of the Systemic Lupus Erythematosus Disease Activity Index; SLICC, Systemic Lupus International Collaborating Clinics; ESR, Erythrocyte Sedimentation Rate.

**Table 2:** Frequencies of genotypes, alleles, and haplotypes of *IL21* gene polymorphisms.

	HC *n* = 300 (%)	SLE *n* = 300 (%)	*p*-Value	OR (95% CI)	*p*_c_-Value
rs2221903 (+3268 T>C)					
TT	258 (86.29)	236 (78.67)	1	-	-
TC	40 (13.38)	58 (19.33)	**0.039**	**1.58 (1.02–2.46)**	0.078
CC	1 (0.33)	6 (2.00)	**0.046** ^†^	**6.56 (1.07–75.60)**	0.092
T	556 (92.98)	530 (88.33)	1	-	**-**
C	42 (7.02)	70 (11.67)	**0.005**	**1.75 (1.17–2.61)**	**-**
Dominant model					
TT	258 (86.29)	236 (78.67)	1	**-**	**-**
TC + CC	41 (13.71)	64 (21.33)	**0.014**	**1.71 (1.11–2.62)**	**-**
Recessive model					
TT + TC	298 (99.67)	294 (98.00)	1	-	-
CC	1 (0.33)	6 (2.00)	0.058 ^†^	0.16 (0.02–1.37)	-
rs2055979 (+1439 C>A)					
CC	90 (30.00)	87 (29.00)	1	-	-
CA	153 (51.00)	154 (51.33)	0.830	1.04 (0.72–1.51)	1
AA	57 (19.00)	59 (19.67)	0.775	1.07 (0.67–1.71)	1
C	333 (55.50)	328 (54.67)	1	-	-
A	267 (44.50)	272 (45.33)	0.772	1.03 (0.82–1.30)	-
Dominant model					
CC	90 (30.00)	87 (29.00)	1	-	-
CA + AA	210 (70.00)	213 (71.00)	0.788	1.05 (0.74–1.49)	-
Recessive model					
CC + CA	243 (81.00)	241 (80.33)	1	-	-
AA	57 (19.00)	59 (19.67)	0.836	0.96 (0.64–1.44)	-
Haplotype ^‡^					
TC	292.13 (48.68)	261.14 (43.50)	1	0.81 (0.64–1.02)	-
TA	263.87 (43.98)	268.86 (44.81)	0.281	1.14 (0.90–1.44)	0.562
CC	39.87 (6.64)	66.86 (11.14)	**0.004**	**1.87 (1.21–2.85)**	**0.008**

^‡^ The haplotype analysis included the rs2221903 (+3268 T>C) and rs2055979 (+1439 C>A) polymorphisms of the IL21 gene. All haplotypes with a frequency <0.03 were excluded from the analysis. Bonferroni correction was applied to the *p*-values to control for multiple comparisons, and the results are shown as corrected *p*-values (*p_c_*-value). The statistical tests used for the allelic and genotype frequencies included the Chi-square test and ^†^ Fisher’s exact test according to the case. A *p*-value < 0.05 was considered statistically significant. The values in bold indicate statistically significant results. Abbreviations: CI, confidence interval; HC, healthy control; OR, odds ratio; SLE, systemic lupus erythematosus.

## Data Availability

The data used to support the findings of this study will be available upon request to the corresponding authors.

## Supplementary Materials

The following supporting information can be downloaded at https://www.mdpi.com/article/10.3390/jcm13154512/s1, Figure S1. The IL-21 levels according to the rs2055979 (+1439 C>A) genotype and rs2055979 and rs2221903 haplotypes in the *IL21* gene. The IL-21 levels according to the rs2055979 (+1439 C>A) genotype (a) and rs2055979 and rs2221903 haplotypes (b) of the *IL21* gene. The data are shown as the median and IQR. The *p*-value was obtained through the Kruskal–Wallis test with Dunn’s post hoc test. IQR: interquartile range.
