# Supplementary material for: High Interleukin 21 Levels in Patients with Systemic Lupus Erythematosus: Association with Clinical Variables and rs2221903 Polymorphism
Source: J Clin Med. 2024 Aug 2;13(15):4512. doi: 10.3390/jcm13154512 (PMC11313274; doi:10.3390/jcm13154512)
Supplement: Supplementary file 1 [file jcm-13-04512-s001.zip › jcm-3129092-supplementary.pdf]

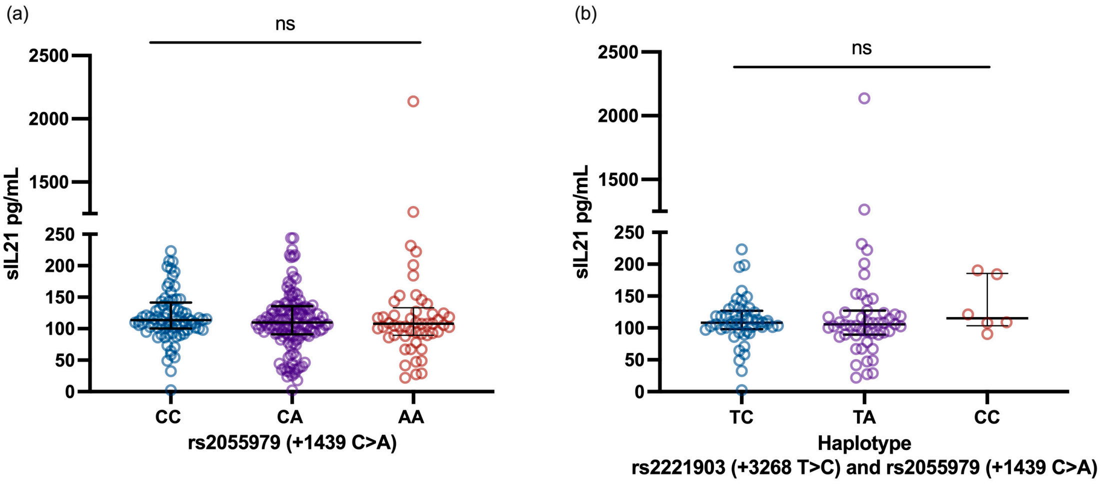

Figure S1. IL-21 levels according to rs2055979 (+1439 C>A) genotypes and rs2055979 and rs2221903 haplotypes in the *IL21* gene. IL-21 levels according to rs2055979 (+1439 C>A) genotypes (a) and rs2055979 and rs2221903 haplotypes (b) of *IL21* gene. Data are shown in median and IQR. p-value was obtained through Kruskal-Wallis's test with Dunn's post hoc test. IQR: interquartile range.
